# Nerve‐Inspired Optical Waveguide Stretchable Sensor Fusing Wireless Transmission and AI Enabling Smart Tele‐Healthcare

**DOI:** 10.1002/advs.202410395

**Published:** 2024-12-04

**Authors:** Tianliang Li, Qian'ao Wang, Zichun Cao, Jianglin Zhu, Nian Wang, Run Li, Wei Meng, Quan Liu, Shifan Yu, Xinqin Liao, Aiguo Song, Yuegang Tan, Zude Zhou

**Affiliations:** ^1^ School of Mechanical and Electronic Engineering Wuhan University of Technology Wuhan Hubei 430070 China; ^2^ School of Information Wuhan University of Technology Wuhan Hubei 430070 China; ^3^ School of Electronic Science and Engineering Xiamen University Xiamen Fujian 361005 China; ^4^ School of Instrument Science and Engineering Southeast University Nanjing Jiangsu 210096 China

**Keywords:** artificial intelligence, hydrogel optical waveguides, stretchable sensors, tele‐healthcare systems, wireless transmission

## Abstract

Flexible strain monitoring of hand and joint muscle movement is recognized as an effective method for the diagnosis and rehabilitation of neurological diseases such as stroke and Parkinson's disease. However, balancing high sensitivity and large strain, improving wearing comfort, and solving the separation of diagnosis and treatment are important challenges for further building tele‐healthcare systems. Herein, a hydrogel‐based optical waveguide stretchable (HOWS) sensor is proposed in this paper. A double network structure is adopted to allow the HOWS sensor to exhibit high stretchability of the tensile strain up to 600% and sensitivity of 0.685 mV %^−1^. Additionally, the flexible smart bionic fabric embedding the HOWS sensor, produced through the warp and weft knitting, significantly enhances wearing comfort. A small circuit board is prepared to enable wireless signal transmission of the designed sensor, thereby improving the daily portability. A speech recognition and human‐machine interaction system, based on sensor signal acquisition, is constructed, and the convolutional neural network algorithm is integrated for disease assessment. By integrating sensing, wireless transmission, and artificial intelligence (AI), a smart tele‐healthcare system based on HOWS sensors is demonstrated to hold great potential for early warning and rehabilitation monitoring of neurological diseases.

## Graphical abstract

This article presents a hydrogel‐based optical waveguide stretchable (HOWS) sensor, an innovative technology for smart tele‐healthcare systems, addressing motor dysfunction in neurological disorders. It focuses on the sensor's design, performance, and applications in monitoring body strain and finger dexterity rehabilitation. The HOWS sensor facilitates gait and gesture recognition, advancing remote health monitoring and rehabilitation efforts effectively.

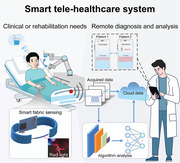

## 1 Introduction

Motor dysfunction is a characteristic symptom of neurological diseases such as stroke and Parkinson's disease.^[^ ^1^, ^2^ ^]^ Flexible strain monitoring of hand, lower limb, and joint muscle movements is an effective method for early detection and quantitative health characterization of this disease. Current research mainly utilizes flexible and stretchable sensors based on the principle of electromagnetic,^[^ ^3^ ^]^ typically use conductive materials such as graphene and carbon nanotubes.^[^ ^4^, ^5^, ^6^, ^7^, ^8^, ^9^, ^10^, ^11^ ^]^ However, challenges related to electromagnetic interference and insufficient biocompatibility restrict their application on complex body surfaces, causing skin inflammation with prolonged use.^[^ ^12^, ^13^, ^14^, ^15^ ^]^ Furthermore, the inherent sensitivity and range limitations of electrical sensors make it difficult to simultaneously accommodate minor muscle tremors and major joint movements, limiting the multi‐point monitoring of the sensors. Sensing‐transmitting integration of fiber optic sensors with anti‐electromagnetic interference provides an excellent candidate for unobtrusive, distributed motion monitoring.^[^ ^16^, ^17^ ^]^ Nevertheless, these sensors often fall short in detecting the wide range of strains associated with complex human activities, from subtle wrist pulses (<1% strain) to significant joint bending (>55% strain).^[^ ^18^ ^]^ How to achieve both high sensitivity and large strain in flexible stretchable sensors has become an urgent problem to be solved.

With the development of advanced materials science, The researchers find hydrogels are particularly well‐suited for monitoring physiological activities due to their low modulus and high flexibility,^[^ ^19^, ^20^, ^21^, ^22^, ^23^ ^]^ yet their strain monitoring is hindered by limited mechanical strength. By combining ionic and covalent polymer networks, the stretchability of the hydrogel can be significantly enhanced, giving it excellent strain‐sensing performance in detecting human activity.^[^ ^24^, ^25^, ^26^, ^27^, ^28^, ^29^ ^]^

With the imbalance in the doctor‐patient ratio, tele‐healthcare systems have become popular because they can obtain data remotely, thereby alleviating pressure on medical resources.^[^ ^30^, ^31^ ^]^ The system is generally required to integrate the sensors into the patient's body seamlessly and monitor physical activities simultaneously.^[^ ^32^, ^33^ ^]^ Most sensor signal receivers need a wired transmission, which restricts daily activities due to cumbersome cables.^[^ ^34^, ^35^ ^]^ Furthermore, experiments have demonstrated that hydrogels exposed to dry environments for extended periods^[^ ^36^ ^]^ may suffer from performance degradation or failure,^[^ ^37^ ^]^ thus limiting their applicability in long‐term disease monitoring.^[^ ^38^, ^39^, ^40^, ^41^ ^]^ Consequently, hydrogel‐based flexible and stretchable sensors lack wide application for clinical early disease warning and rehabilitation monitoring. Additionally, most of the proposed sensors primarily focus on monitoring physical data that still necessitate doctors' expertise for evaluation and judgment. The availability of tools capable of objective and autonomous data analysis has the potential to enhance the efficiency of medical diagnosis. Therefore, artificial intelligence (AI) signal analysis and pattern recognition have emerged as effective methods for diagnosing and providing early warnings of diseases.^[^ ^42^, ^43^, ^44^, ^45^ ^]^ Moreover, sensors can serve as a channel for human‐machine interaction in medical environments,^[^ ^46^ ^]^ and can also be combined with other innovative technologies to form sensing systems for use in medical environments.^[^ ^47^ ^]^ Machine learning, such as support vector machines,^[^ ^48^ ^]^ can analyze signals, objectively assess a patient's disease and rehabilitation status, and aid in early disease warning. Therefore, the integration of sensing, transmission, and AI evaluation functions has become an inevitable development trend for flexible and stretchable sensors.

Inspired by the information transmission mechanism in nerve fibers, this paper develops a wearable hydrogel‐based optical waveguide stretchable (HOWS) sensor. The hydrogel fiber component of the sensor applies a double network (DN) structure, the strain detection range is set between 0% and 100%, exhibiting high sensitivity (0.685 mV %^−1^) and stability (2,000 strain cycle tests), enabling distributed strain monitoring during human activities. To meet daily monitoring needs, the HOWS sensor's performance is maintained for at least 24 h without significant attenuation. A compact circuit board was designed for the real‐time wireless acquisition of strain signals, enhancing the device's portability. Furthermore, the proposed sensor has been embedded by warp‐weft knitting to form a flexible and smart bionic fabric. A speech recognition system and a human‐machine interaction system were developed based on data obtained from HOWS sensors, providing new ideas for patient communication and rehabilitation. To improve diagnostic efficiency, the HOWS sensor is integrated with AI algorithms to accurately predict abnormal gait signals and provide early disease warning. The intelligent tele‐healthcare system fuses HOWS sensors, wireless signal transmission, and AI algorithms, suited for multiple scenarios such as human‐machine interfaces (HMI), disease warning, and patient rehabilitation monitoring.

## 2 Design, Function, and Application of the HOWS Sensor

**Figure 1:**
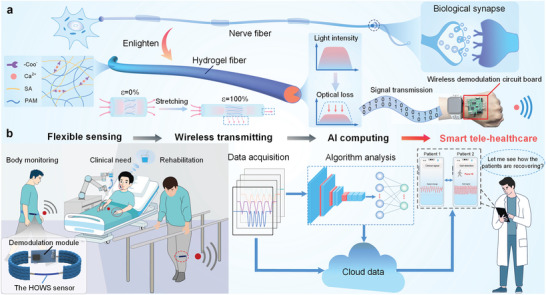
Design, function, and application of the HOWS sensor. a) The inspiration diagram, concept diagram, and sensing principle diagram of the hydrogel flexible optical fiber waveguide. The components of the hydrogel are marked in the figure, and the actual image of the developed demodulation circuit board is in the lower right corner. b) Concept diagram and application scenarios of integrated sensing, wireless transmission, and AI computing.

In nerve fibers, signals are conducted through neurons and ultimately transmitted via synapses.^[^ ^49^ ^]^ Inspired by this model, the HOWS sensor is proposed in **Figure** **1**a. A smart tele‐healthcare system integrating sensing, wireless transmission, and AI computing based on the HOWS sensor is constructed, which can be used for wearable monitoring, HMI, and medical disease monitoring (Figure 1b).

The PAM/SA‐Ca^2+^ DN structure of the hydrogel fiber enhances the mechanical strength and performance of the HOWS sensor. The inclusion of an appropriate amount of glycerol further improves its resistance to drying. The generated optical signal is transmitted through a plastic optical fiber and passes through the hydrogel fiber. When strain is applied, the intensity of the optical signal within the hydrogel fiber decreases, following the Beer–Lambert law:^[^ ^50^ ^]^

(1) A=ECL

where *A* represents the substance's absorbance, *E* is the material's molar absorptivity, *C* is the concentration of the attenuating chemical in the medium, and *L* is the optical path length. When the hydrogel fiber waveguide is stretched from its initial length to ε ($\varepsilon =\frac{L-l_{0}}{L_{0}}$, *l*_0_ is the initial length of the hydrogel fiber), the light intensity loss is calculated using the equation:

(2) $$A_{\left(\varepsilon \right)}=\;ecl_{0}\varepsilon +D_{\left(\varepsilon \right)}$$

where *e* represents the hydrogel's light absorption rate, and *c* denotes the material concentration, both remaining constant during stretching. *D*_(ε)_ accounts for the coupling loss at the hydrogel's connection points in its stretched state. During stretching, the hydrogel fiber's light intensity loss is positively correlated with the strain magnitude.

The HOWS sensor can accurately capture the strain information generated by the activities of human muscles and joints, detecting various movements of the human body, and can also be integrated into warp and weft woven fabrics to achieve comfortable wearable sensing of smart fabrics. Connected to the designed small demodulation circuit board, the collected sensor data can be wirelessly transmitted to the host computer, effectively integrating sensing and wireless transmission. For the data stored therein, neural network analysis can be employed to further classify the data, or the collected signals can be displayed in real‐time on the interface through the designed mobile phone application to achieve remote monitoring. These applications contribute to the advancement of smart tele‐healthcare.

## 3 Preparation and Signal Reception of the HOWS Sensor

**Figure 2:**
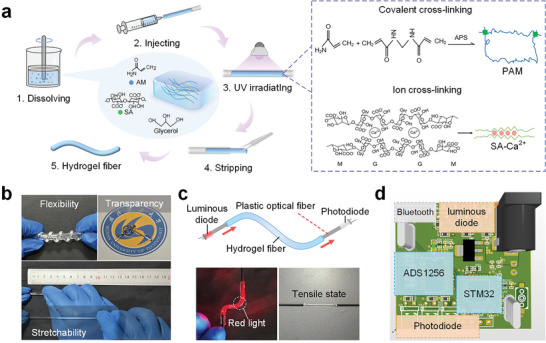
Preparation and signal reception of the HOWS sensor. a) Preparation process and reaction principle of hydrogel fibers. b) Testing of hydrogel fibers (flexibility, transparency, stretchability). c) Structure of the HOWS sensor with energized light, the inset shows the HOWS sensor in the energized infrared light and the stretched state. d) Components of various parts of the optical signal demodulation circuit board.

The hydrogel fiber component of the sensor was synthesized using a one‐pot method, which was divided into several straightforward steps. First, sodium alginate (SA), acrylamide (AM), N, N’‐methylenebisacrylamide (MBA), ammonium persulfate (APS), and glycerol were homogeneously mixed with an aqueous solution containing 0.5 mL of CaCl_2_ solution at room temperature until completely dissolved. After degassing, the mixture was injected into a silicone tube mold using a syringe and then polymerized under UV light. The SA and AM mixtures, crosslinked with MBA and initiated by APS, were polymerized under UV light to form a covalent polyacrylamide (PAM) network, followed by the formation of a secondary network with linear sodium alginate in the presence of divalent cations (**Figure** **2**a). Hydrogen bonding between the amine groups of PAM and the hydroxyl groups of SA enhanced the intermolecular strength. After the polymerization was complete, the fibers were removed from the silica tubing, and the residue was washed away, yielding transparent and elastic hydrogel fibers with excellent flexibility, transparency, and stretchability (Figure 2b). By adjusting the length and diameter of the silicone tube, the size of the prepared PAM/SA‐Ca^2+^ DN hydrogel fibers was tailored to meet different preparation requirements.

Due to the hydrogel's water content, it tends to dehydrate in dry environments, leading to reduced stretchability and sensor functionality, as observed in fibers lacking water‐retention mechanisms. Incorporating glycerol has been shown to improve the fiber's resilience to drying (Figure S1, Supporting Information), thereby prolonging the operational lifespan of the HOWS sensor.^[^ ^51^ ^]^ The mass change of the hydrogel fiber over 24 h serves as an indicator of its water‐retaining capacity across varying glycerol concentrations (Figure S2, Supporting Information). Test results show that hydrogel fibers with a 25% glycerol concentration maintain their mechanical stretchability over prolonged periods under dry storage conditions. However, excessive glycerol content adversely affects the dissolution of SA in the solution. Adjusting the AM content in the hydrogel double network alters its mechanical properties (Figure S3, Supporting Information). Incorporating SA into the secondary network enhances the material's resilience, influencing fiber swelling (Figure S4, Supporting Information) and hysteresis (Figure S5, Supporting Information). Test results indicate that fibers with 25% AAM and 0.2 g SA exhibit the most favorable mechanical properties. The maximum tensile strain of the prepared fiber can reach 600%.

During the polymerization of the hydrogel fiber, a wire stripper was employed to remove 1 cm of protective coating from the front ends of two plastic optical fibers. The ends of the silicone sleeve were inserted to align the centers of the optical fiber and the sleeve. After polymerization under ultraviolet light, the sleeve was extracted, and the fiber underwent cleaning with deionized water to eliminate surface residues, resulting in the HOWS sensor (Figure 2c). Different diodes were connected to the ends of the sensor, with a luminous diode emitting red light. Upon passing through the sensor, the signal was received by a photodiode and integrated into a compact optical signal demodulation circuit board (Figure 2d). When the HOWS sensor is stretched by an external force, internal light intensity loss increases, detected by the demodulation circuit board, and transmitted via Bluetooth wireless to the host computer. This development integrates the HOWS sensor with a wireless transmitting module.

## 4 Performance and Characteristics of the HOWS Sensor

The sensing performance of the HOWS sensor is dependent on the diameter and length of the hydrogel fiber. The tensile properties of hydrogel fibers with various diameters (Figure S6, Supporting Information) and lengths (Figure S7, Supporting Information) were investigated. The experimental results indicate that optimal tensile properties were observed with a hydrogel fiber of 3 mm in diameter, while the highest sensitivity was achieved at a length of 2 cm. Consequently, a hydrogel fiber with a diameter of 3 mm and a length of 2 cm was selected for the sensing component. Once the sensor size was determined, it was calibrated across a range from 0% to 100%, with increments of 10%.

**Figure 3:**
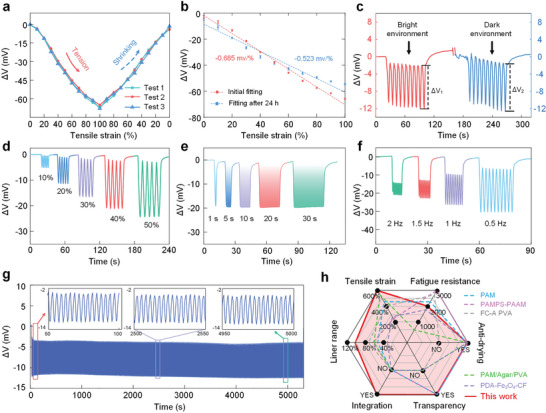
Performance and characteristics of the HOWS sensor. a) Static tensile calibration of hydrogel fiber with 10% step size at 100% strain range. b) Calibration fit straight line before and after 24 h water loss of the sensor. c) Equal strain tensile testing of the HOWS sensor in dark and light environments. d) Transducer variable amplitude (10–50%) signal testing. e) Transducer variable time (1–30 s) signal testing. f) Transducer variable frequency (0.5–2 Hz) signal testing. g) Durability test of the HOWS sensor up to 2,000 cycles. f) Performance comparison chart of wearable sensors for strain detection.^[^ ^52^, ^53^, ^54^, ^55^, ^56^ ^]^

Varying degrees of mechanical deformation can be effectively converted into discernible signals with high fidelity. According to the calibration, it can be seen that the sensitivity of the HOWS sensor is 0.685 mV %^−1^ with a good linearity of R^2^ = 0.989. The hysteresis and repeatability errors are 9.401% and 2.869%, respectively (**Figure** **3**a; Table S1, Supporting Information). The sensor maintained excellent strain sensitivity even after 24 h, despite slight water loss from the hydrogel fiber (Figure 3b). Environmental tests conducted under both light and dark conditions revealed that the strain detection capability of the HOWS sensor was minimally impacted by natural light, as indicated by nearly identical voltage changes (ΔV_1_≈ΔV_2_) (Figure 3c), affirming its suitability for all‐weather wearable applications. Further testing validated the strain‐sensing capabilities of the HOWS sensor, including response time (Figure S8, Supporting Information) and stability during incremental stretching (Figure S9, Supporting Information). In addition, the HOWS sensor was calibrated from 0 to 90 degrees to adapt to more complex strain environments (Figure S10, Supporting Information). Dynamic tests, including reciprocating stretching from 10% to 50%, and variations in time (1–30 s) and frequency (0.5–2 Hz) at a 50% stretching rate, demonstrated the responsiveness of the HOWS sensor to dynamic changes (Figure 3d–f). A fatigue test with a 40% stretching amplitude confirmed the enduring performance of the HOWS sensor after 2000 cycles (Figure 3g), highlighting its durability and adaptability to various environments. Comparative analysis with previous hydrogel‐based sensors underscored the superior performance of the HOWS sensor in terms of tensile strain, fatigue resistance, anti‐drying, transparency, integration, and liner range (Figure 3h), making it highly effective for monitoring human activities.

## 5 The HOWS Sensor for Strain Monitoring in Different Parts of the Human Body

**Figure 4:**
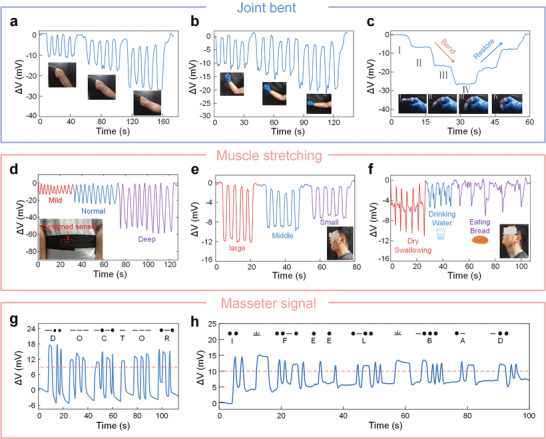
The HOWS sensor for strain monitoring in different parts of the human body. a) Signal test at different elbow extension angles. b) Signal test at different finger joint extension angles. c) Signal test at different knee joint extension angles. d) Breathing test at different levels. e) Signal test for different levels of masseter muscle. f) Swallowing test with different throat conditions (dry swallow, drinking water, eating). g,h) Masseter muscle signal speech translation system, including (g) word test based on Morse code and (h) sentence test.

Human skin is soft and elastic, and the low modulus and excellent flexibility of hydrogel fibers enable them to fit well on the complex surface of the skin. Therefore, the HOWS sensor can detect strain by adhering to the surface of human skin. In order to verify the practicality of the HOWS sensor, the performance of the sensor was tested at different bending angles of the knee, elbow, and finger (**Figure** **4**a–c). The results show that the HOWS sensor can excellently detect large strain signals generated by human joint movements. When it is specifically applied to finger joints to simulate writing, the sensor shows excellent repeatability for the same letter, and there is a clear distinction between different letters (Figure S11, Supporting Information). In addition to the signal testing of different joints, the stretching of muscles was also tested. The HOWS sensor was fixed to the abdomen to simulate different degrees of deep breathing. For larger muscle stretches, the HOWS sensor can be sewn into an elastic waistband to collect signals from different breathing levels (Figure 4d). In addition, after the sensor is sewn into the fabric to make smart fabric, it can also collect breathing signals of different levels (Figure S12, Supporting Information). This smart fabric simplified the installation process and improved comfort, achieving daily comfort wear needs.

For small muscle stretching, the sensor was taped to the masseter muscle on the face, simulating different degrees of mouth opening and closing movements, which the HOWS sensor could distinguish well (Figure 4e). It is crucial to monitor patients with dysphagia and identify potential choking risks during meals. Therefore, the sensor was taped to the throat, simulating throat movements during dry swallowing, drinking water, and eating, and could be well distinguished (Figure 4f), with the generated signal having multiple peaks and a long duration. In order to take into account both indoor and outdoor monitoring, the HOWS sensor was tested for signal measurements in different environments. The results showed that the signals collected by the sensor in different environments were similar, showing good environmental adaptability (Figure S13, Supporting Information). An HMI interface was developed using the masseter muscle signal (Figure S14, Supporting Information), and a speech recognition system driven by masseter muscle movement was finally formed using the masseter muscle signal features. The system converts different degrees of mouth movement into recognizable digital signals based on the duration of the movement. These digital signals are combined with Morse code to form an effective communication bridge, converting muscle activity into meaningful language (Figure 4g,h; Videos S1 and S2, Supporting Information). This is especially important for patients with speech or language disorders. In addition, a simplified decoding chart for clinical patients was developed (Figure S15, Supporting Information) to make it easier for patients to express their daily needs. This system offers great potential for patient care applications.

## 6 Finger Dexterity Rehabilitation Exercises Based on Mechanical Devices

**Figure 5:**
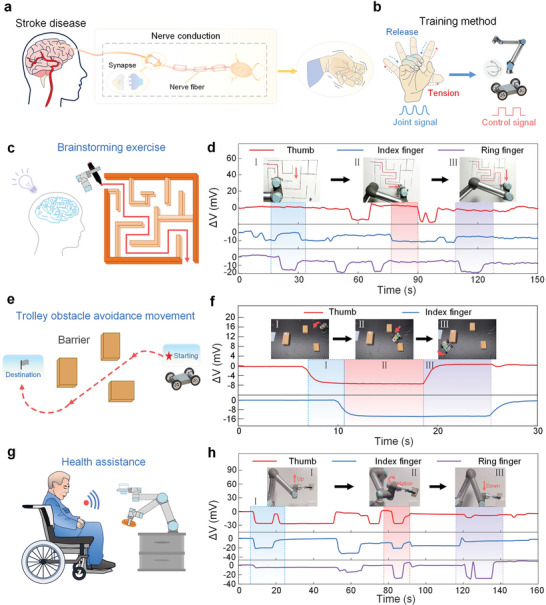
Finger dexterity rehabilitation exercises based on mechanical devices. a) Comparison of finger joint rehabilitation training before and after stroke patients. b) Two rehabilitation methods designed for finger joint rehabilitation training. c) Brainstorming exercises based on robotic arm control. d) Signals when the robotic arm moves in different directions. e) Path planning for obstacle avoidance exercises for the control car. f) Signal changes of the car during actual movement. g) Finger control experiment based on robotic arm control. h)Signals during robotic arm movement.

Stroke is a high‐risk disease that causes damage to brain nerve tissue, thereby blocking nerve transmission,^[^ ^57^ ^]^ and seriously threatening human health. This can lead to motor and cognitive impairments, as well as emotional distress, which seriously affects the patient's life. Robot‐assisted therapy is effective in improving upper limb motor function, learning, and memory in stroke patients, and the possible mechanism involves improving neuroplasticity. By moving the numb limbs, patients can enhance the brain's perception of movement, stimulate damaged nerve cells to form new neural circuits, and restore motor function (**Figure** **5**a).^[^ ^58^, ^59^ ^]^

This work proposes a finger joint rehabilitation system that uses motion‐control medical devices. Patients can manipulate the robotic arm or control the car by wearing HOWS sensors. In the test, the hydrogel optical fiber on the finger joint captures the bending motion and converts it into an electrical signal transmitted via Bluetooth to control the movement of the UR5 robotic arm and the cart (Figure 5b). The movement of the robotic arm is controlled by combining signals from three fingers into five gestures; the planar movement of the cart is controlled by four gestures, which are consistent with the direction of the robotic arm (Figure S16, Supporting Information). First, a “brainstorming” experiment was designed (Figure 5c and Video S3, Supporting Information). By wirelessly controlling the robotic arm to complete a simple maze, spatial awareness, hand‐eye coordination, and attention can be improved. Each change in the steering of the robotic arm was clearly recorded (Figure 5d). In addition, a fixed track movement test and a car obstacle avoidance test were designed to guide patients to wirelessly control the car (Figure 5e, Figure S17, and Video S4, Supporting Information). The signals of different finger bending were also recorded to control the movement of the car (Figure 5f). Furthermore, a feeding experiment based on a robotic arm was carried out to simulate patients manipulating the robotic arm to drink water and eat in order to enhance self‐care, limb coordination, and motor control (Figure 5g; Figure S18, Supporting Information). By recording the signal changes, it can be seen that the signal will produce corresponding changes every time the control operation is performed, and it will be transmitted to the robotic arm for real‐time control to achieve the purpose of eating and drinking (Figure 5h; Video S5, Supporting Information,).

The above experiments achieve the coexistence of rehabilitation and data supervision through the coordination of task configuration, data supervision, and visual feedback. On the one hand, with the help of task practice and human‐machine interaction, the patient's sense of immersion is enhanced and the boredom of deliberate practice is avoided; on the other hand, scenario‐based real‐time data collection improves the dimension and reliability of rehabilitation evaluation. This system has potential future applications in controlling smart wheelchairs and other medical devices, so that smart medical devices can better serve patients and assist patients in completing complex physiological movements. In conclusion, the finger joint rehabilitation system provides a diverse and effective approach to stroke rehabilitation, supporting a range of patient needs through interactive and efficient training tasks.

## 7 Gait and Gesture Recognition and Remote Monitoring Application

**Figure 6:**
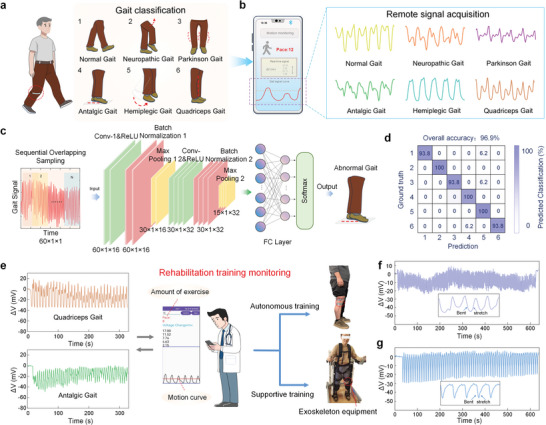
Gait and gesture recognition and remote monitoring application. a) Differentiation of abnormal gait features b) Mobile phone application captures abnormal gait signal features. c) Gait signal classification and processing flow based on the 2D convolution algorithm model. d) Confusion matrix of gait signal classification and recognition results. e) HOWS sensor is used in conjunction with a mobile phone application for assisted walking exercise and autonomous monitoring. f) Signal changes during autonomous exercise. g) Signal changes during machine‐assisted exercise.

Walking is a complex cyclical process that requires close coordination between the brain and body parts. Lesions in certain parts of the body can lead to abnormal gait patterns, often indicating early neurological and musculoskeletal diseases.^[^ ^60^, ^61^ ^]^ Abnormal gait formation may be caused by central nervous system damage, weakness of specific muscle groups, or lower limb pain. These diseases affect the muscles, joint movements, and neural control during walking, which can lead to different gaits such as neuropathic gait: lifting the leg to prevent foot‐dragging, festinating gait: characterized by short steps, foot‐dragging, and leaning forward, antalgic gait: slight lean toward one side, with external rotation and bending of the affected lower limb to avoid heel strike, hemiplegic gait: where the affected limb rotates outward, making the walking path circular, and quadriceps gait: walking with an extended hip joint and passive knee extension causing hyperextension of the knee (**Figure** **6**a). Research and analysis of these gait abnormalities can provide reliable data support for disease diagnosis.

In the actual test, the HOWS sensor was attached to the outside of the knee joint to artificially simulate various abnormal gaits, and the remote signal acquisition was performed through the developed mobile phone APP (Figure 6b, Figure S19, and Video S6, Supporting Information). Since each abnormal gait has its special posture and limb direction, each abnormal gait has its specific signal waveform. To further distinguish gait signals, a convolutional neural network was used to classify and analyze the collected data (Figure 6c). The convolutional neural network (CNN) is capable of automatically extracting local features from time series data and capturing complex features from low level to high level. It is particularly well‐suited to the processing of a diverse array of collected gait signals. The results showed that the accuracy of the gait signal recognition model based on a CNN reached 96.9% (Figure 6d), which is better than the relevant algorithms and also highlights the potential of HOWS sensors in early disease detection (Table S2 and Figure S20, Supporting Information). Moreover, neurological diseases such as stroke and Parkinson's disease may cause speech disorders in patients, and the gestures of patients become a key way for doctors to understand the patients' care needs, dietary needs, and other requirements. Therefore, the signals of the thumb, middle finger, and little finger under different designs of medical gestures were collected, and the collected data were classified using AI technology, with a recognition accuracy of up to 95.7% (Figure S21, Supporting Information).

In rehabilitation, patients with abnormal gait benefit from physical exercise to improve muscle relaxation and trunk control.^[^ ^62^ ^]^ Mild patients enhance motor balance through autonomous walking exercises, aiding in the restoration of a normal walking rhythm. For those with limited mobility, medical devices such as exoskeletons provide support during gait correction exercises. Monitoring movement trajectories via smartphones enables healthcare providers to better understand patients’ joint activities, facilitating the development of targeted exercise plans (Figure 6e‐g). As the patient's condition progresses, the gait monitoring system evolves into a health tool that records daily movement, assisting doctors in evaluating rehabilitation and promptly adjusting training plans (Figure S22 and Video S7, Supporting Information). Switching between different interfaces in the APP allows for the monitoring of various signals or simultaneous observation across multiple mobile APPs (Video S8, Supporting Information). By integrating sensing, wireless transmission, and AI computing, a comprehensive smart tele‐healthcare system has been developed. This real‐time monitoring and evaluation system is designed to shorten recovery periods, streamline the diagnosis and treatment process, and offer patients a more scientific, convenient, and effective path to recovery.

## 8 Discussion

This work introduces a hydrogel‐based optical waveguide stretchable (HOWS) sensor, inspired by the information transmission mechanism found in nerve fibers. The innovative sensor integrates wireless transmission and AI computing capability for the advanced smart tele‐healthcare system. The system is designed to monitor human physiological activities, facilitate rehabilitation, and identify abnormal gait patterns, therefore significantly improving disease warning and rehabilitation monitoring.

The HOWS sensor is constructed from a hydrogel fiber featuring a PAM/SA‐Ca^2+^ DN structure, with plastic optical fibers situated at both ends. The relationship between the optical signal intensity and strain is characterized according to the Beer‐Lambert law. To optimize performance parameters, the mechanical properties of the hydrogel fibers were meticulously examined under various conditions. The HOWS sensor can continuously monitor human physiological activities for a minimum of 24 h with glycerol mixing. In sensor performance tests, the HOWS sensor demonstrated high sensitivity (0.685 mV %^−1^), a broad strain detection range (up to 100%). The stretch rate is much higher than that of sensors made of silicon optical fiber.^[^ ^63^ ^]^ In tensile cycle tests, the HOWS sensor demonstrated strong fatigue resistance for at least 2,000 cycles. The hydrogel fibers’ low modulus and high flexibility ensure that the HOWS sensor conforms well to complex skin surfaces, enabling precise monitoring of strain signals across various body joints, including the knees, elbows, and fingers. Inspired by the growth of human nerves in tissues, sensors are embedded in the human body through warp and weft knitting technology to form a flexible smart bionic fabric. Compared to methods such as adhesives or structures, the fabric simplifies the installation process, improves comfort, and meets the need for comfortable daily wear. The compact demodulation circuit board supports wireless transmission of the acquired sensor signals, in contrast to the wired sensing modes and bulky demodulation devices,^[^ ^64^, ^65^ ^]^ this design significantly enhances both comfort and portability for the sensor.

The HOWS sensor demonstrates outstanding strain‐sensing capabilities, environmental adaptability, and wireless transmission, making it highly promising for human activity monitoring. These sensors offer substantial potential for integration into smart tele‐healthcare systems, advancing medical rehabilitation and early disease warning capabilities. The existing research results of flexible sensors mainly focus on the detection of human body parameters, lacking synchronous analysis capabilities such as disease diagnosis,^[^ ^66^, ^67^ ^]^ The HOWS sensor proposed in this work not only detects signals from different parts of the human body, but also develops a speech recognition system based on masseter signals which have been developed to facilitate effective communication for patients with speech or language impairments. Leveraging the stretchability of the sensor and wireless transmission capabilities, a human‐machine interaction system has also been developed to provide patients with a variety of recovery methods by controlling the robotic arm to complete maze games and daily activities or controlling the car to achieve trajectory control. For patients with abnormal gaits, the collected data is processed and classified by CNN, and the recognition accuracy reaches 96.9%. High recognition accuracy can also be achieved for different designed medical gestures to facilitate patients' daily medical needs. The developed mobile application supports remote monitoring of patients with abnormal gaits, enhancing rehabilitation management and enabling adjustments to recovery intensity to improve patient outcomes. By integrating AI, the HOWS sensors allow for accurate disease identification and prediction, paving the way for early and personalized treatment plans.

In conclusion, the HOWS sensor represents a powerful tool for disease diagnosis and healthcare, with ongoing exploration aimed at real‐world applications. In the future, further optimization of the circuit board size and combination of the self‐powered function will enable the optimization of certain components, thus reducing the size of the demodulation circuit board and improving portability. Future developments in self‐powered sensors will enhance the capabilities for wireless monitoring, potentially enabling innovations such as remote wheelchair control based on wireless transmission, and advancing human‐machine interaction in intelligent medical care.

## 9 Experimental Section

### Preparation of Hydrogel Fiber and the HOWS Sensor

The hydrogel fiber was synthesized through a “one‐pot method” with the following procedure: Reagents were precisely measured using a high‐precision electronic balance (model: Delixi high‐precision electronic balance, CZ0002). Initially, SA (0.2 g) powder was dissolved in deionized water (10 g), then 0.5 wt.% CaCl_2_ solution was added (0.5 mL). The mixture was stirred at a constant speed using a magnetic stirrer at room temperature until the SA was fully dissolved. Subsequently, AM (5 g), APS (0.015 g), and MBA (0.005 g) were incorporated. A glycerol aqueous solution (5 g) was then gradually injected using a syringe, with slow stirring to ensure a uniform mix. The solution, free of bubbles, was stored in the dark to prevent premature polymerization.

The SA‐PAM precursor solution containing Ca^2+^ ions was carefully injected into a silicone sleeve mold using a syringe, and the polymerization was initiated under ultraviolet light for 20 min. After the polymerization process was complete, the fiber was carefully removed from the silicone tube and rinsed to eliminate any surface residues.

During the assembly of the HOWS sensor, a wire stripper was used to remove the outer cladding from the front 1 cm of two plastic optical fibers. These optical fibers were then inserted into the two ends of the partially polymerized hydrogel fiber, ensuring precise center alignment. Following the completion of polymerization, the silicone sleeve was removed, resulting in the final HOWS sensor.

### Mechanical Property Test of Hydrogels

Hydrogel fibers with different diameters and different acrylamide contents were prepared, using SRI (Sunrise Instruments Nanning 530 007, China; 45 499 Irvine Dr. Novi, MI48374, USA, M3705C) to carry out tensile tests with a step size of 100%. The relationship between the fiber length and the magnitude of the force applied to it was recorded.

### Swelling Test of Hydrogels

Prepare hydrogel fibers with a diameter of 3 mm and a length of 5 cm and measure their mass (W_d_) at room temperature, immerse them in a deionized water solution, and examine the change in mass of the hydrogel fibers within 48 h. The weight of the tester was measured at intervals of 4 h (W_s_), and the rate of swelling of the hydrogel (Q) is given by the following equation:

(3) $$Q=\frac{w_{s}-w_{d}}{w_{d}}\;\times 100\%$$

where: W_d_ – the weight of hydrogel fiber in an initial state, W_s_ – the weight of hydrogel fiber at a specific time

### Anti‐Drying Test of Hydrogels

Prepare hydrogel fibers with different glycerol contents of 3 mm in diameter and 5 cm in length, measure their initial mass in a dry environment, place the hydrogel in a dry environment, and examine the mass change of the hydrogel fibers every 4 h, meanwhile, calculate the weight loss ratio of the hydrogel fibers after 24 h, and the calculation formula is the same as that of the swelling test.

### Mechanical Hysteresis Test of Hydrogels

Hydrogel fibers, each 5 cm in length and with varying SA contents, were prepared and subjected to cyclic tensile testing. The fibers were fixed on a stepper motor (Beijing Times Chaoqun Electrical Technology Co., Ltd., CBX1024‐400) and tested under strain levels of 50%, 100%, and 200% to evaluate the length of the fibers before and after stretching.

### Demodulation Circuit Board Design and Signal Transmission Mechanism

The HOWS sensor incorporated a demodulation module housed within a 10 cm square integrated circuit board, which was optimized for wearable applications and comparable in size to a standard wristwatch. This board was outfitted with light‐emitting diodes and photodiodes for signal transmission and reception, as well as a Bluetooth module for data communication. Signal emission was achieved using three SFH756 diodes, which coupled light signals into the sensing fiber. On the opposite end of the fiber, SFH250 photodiodes captured the altered light signals after they had traversed the hydrogel fibers, converting them into electrical signals. These signals were then digitized by the ADS1256 chip embedded within the processing unit and subsequently processed by the STM32 chip. The processed data was transmitted wirelessly to mobile devices, such as computers and smartphones, via Bluetooth.

### Gait and Gesture Signal Classification Processing Based on 2D Convolutional Neural Networks

Overlap sampling was conducted on the collected data of gait signals and gesture signals, with a sample length of 50 and a step size of 10, resulting in 456 data sets and 1408 data sets, respectively. The data was then divided into a training set and a test set in an 8:2 ratio. The training set was used to train the CNN model to determine the optimal model parameters. This trained model was subsequently employed to predict the outcomes of the test set, and the predictions were used to evaluate the model's performance. The input to the model was the voltage signal, while the output was the gait category label. The network architecture included an input layer, two convolutional layers, two batch normalization layers, two ReLU activation layers, two max‐pooling layers, one fully connected layer, and one Softmax layer. The training was conducted using the Adam optimizer, with a piecewise decaying learning rate strategy that enabled dynamic adjustments to the learning rate throughout the training process.

## Conflict of Interest

The authors declare no conflict of interest.

## Author Contributions

T.L. conceived the idea. T.L., Q.W., and Y.Z. designed and carried out the experiments. Z.C., J.Z., and R.L. assisted with experiment operations. Q.W., Y.Z., and Z.C. analyzed data. Q.W. and Y.Z. wrote the paper. N.W., S.Y., X.L.,W.M., A.S., Y.T., and Z.Z. revised the paper. All authors discussed the results and commented on the manuscript.

## Supporting information



Supporting Information

Supplemental Video 1

Supplemental Video 2

Supplemental Video 3

Supplemental Video 4

Supplemental Video 5

Supplemental Video 6

Supplemental Video 7

Supplemental Video 8

## Data Availability

Research data are not shared.
