# Supplementary material for: Nerve‐Inspired Optical Waveguide Stretchable Sensor Fusing Wireless Transmission and AI Enabling Smart Tele‐Healthcare
Source: Adv Sci (Weinh). 2024 Dec 4;12(4):2410395. doi: 10.1002/advs.202410395 (PMC11789582; doi:10.1002/advs.202410395)
Supplement: Supplementary file 1 — Supporting Information [file ADVS-12-2410395-s009.docx]

Supporting Information for

**Nerve-Inspired Optical Waveguide Stretchable Sensor Fusing Wireless Transmission and AI Enabling Smart Tele-healthcare**

*Tianliang Li^1^, Qian’ao Wang^1*^, Zichun Cao^1^, Jianglin Zhu^1^, Nian Wang^1^, Run Li^1^, Wei Meng^2^, Quan Liu^2^ , Shifan Yu^3^, Xinqin Liao^3*^, Aiguo Song^4*^, Yuegang Tan^1^, Zude Zhou^1^*

**Affiliations:**

^1^School of Mechanical and Electronic Engineering, Wuhan University of Technology, Wuhan, Hubei, 430070, China.

^2^School of Information, Wuhan University of Technology, Wuhan, Hubei, 430070, China.

^3^ School of Electronic Science and Engineering, Xiamen University, Xiamen, Fujian, 361005, China.

^4^School of Instrument Science and Engineering, Southeast University, Nanjing, Jiangsu, 210096, China

Table S1. Related parameters of the HOWS sensor

| Type of sensor | Sensitivity | linearity | Hysteresis error | Repeatability error | Measuring range |
| --- | --- | --- | --- | --- | --- |
| The HOWS sensor | 0.685 mV %^-1^ | R^2^ = 0.985 | 9.401% | 2.869% | 0%-100% |

Table S2. Recognition accuracy results of different algorithms

| Algorithm used | Accuracy | F1 Score | Model prediction time |
| --- | --- | --- | --- |
| Extreme Learning Machine (ELM) | 75.0% | 0.75 | 0.0045 s |
| Back Propagation Neural Network (BPNN) | 80.2% | 0.81 | 0.0074 s |
| Random Forest (RF) | 93.8% | 0.94 | 0.0140 s |
| This work (CNN) | 96.9% | 0.97 | 0.0152 s |


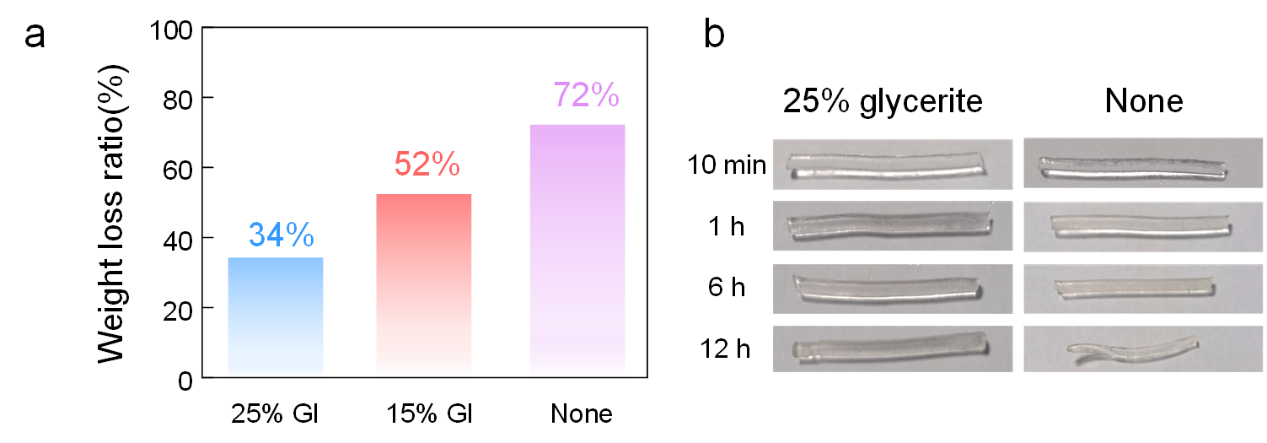


**Figure S1.** Tests on drying resistance of hydrogel fibers at different glycerol contents. a) Water loss characterization of hydrogel fiber under a dry environment within 12 h, hydrogel fiber with 25% glycerol content still has excellent mechanical tensile property after being stored in a dry environment for 12 h, while glycerin-free hydrogel changes into a dry phase and loses mechanical tensile property after 12 h. b) Water loss ratio of hydrogel fiber with different glycerol content after 24 h.

**
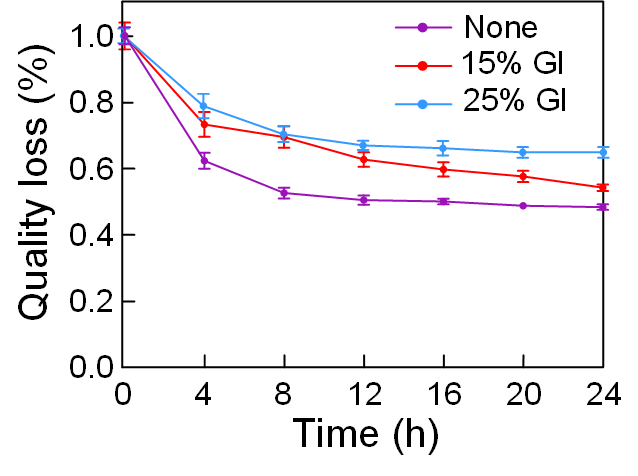
**

**Figure S2.** Statistics of mass loss percentage of hydrogel fibers after 24 hours under different glycerol ratios.

**
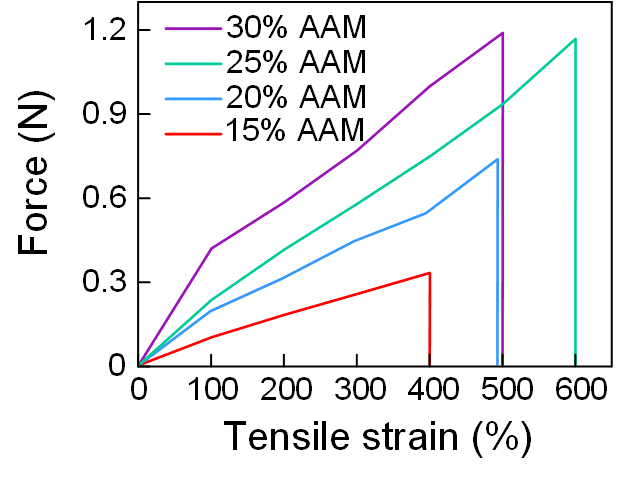
**

**Figure S3.** Mechanical tensile test of hydrogel fibers with different AAM contents.


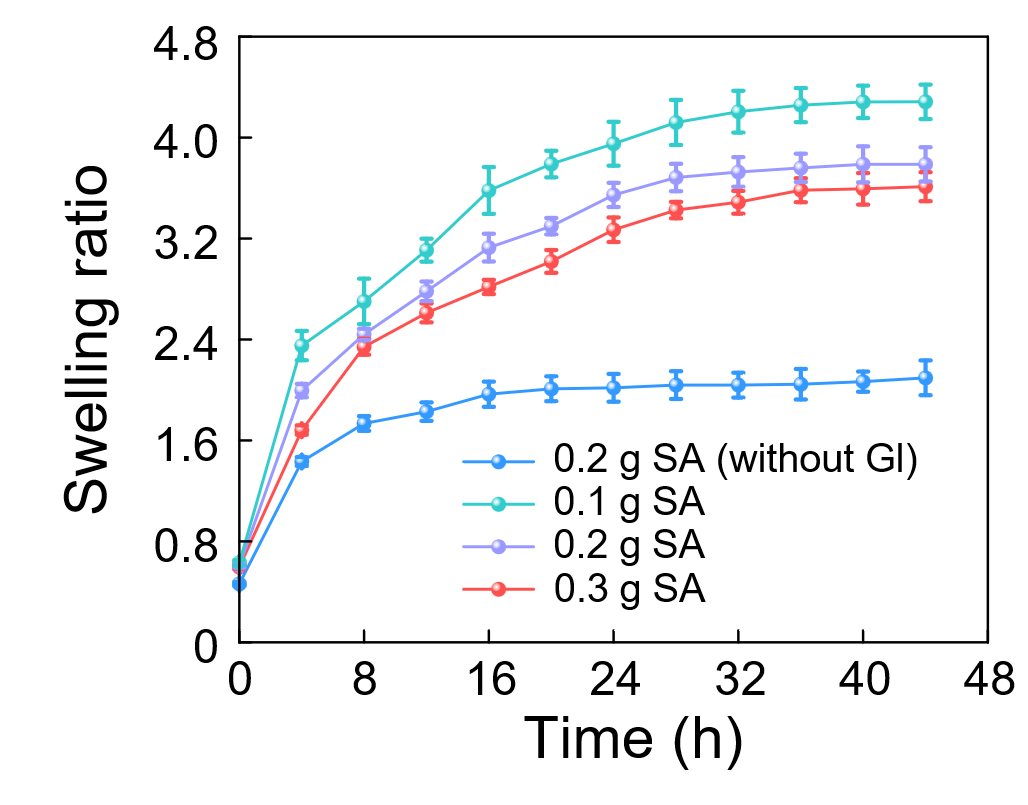


**Figure S4.** Statistical changes of the swelling ratio between sodium alginate added with different contents (0.1 g, 0.2 g, 0.3 g) and glycerol aqueous gel fibers, in which the change of sodium alginate added with 0.2 g without glycerol was taken as the control.


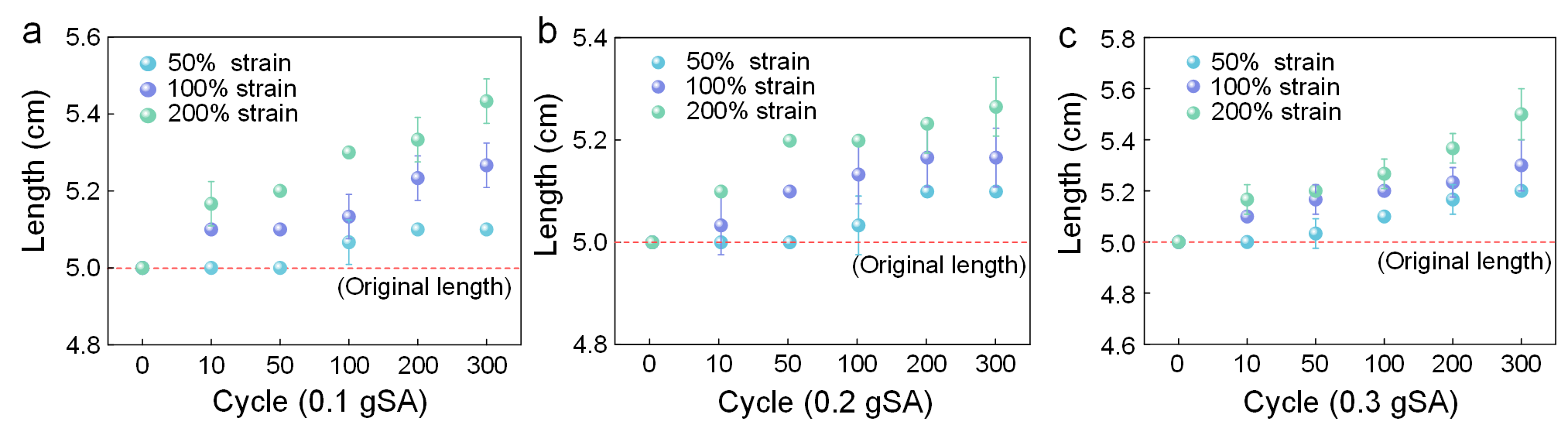


**Figure S5.** Mechanical hysteresis test of underwater gel fiber with different sodium alginate content. a) 0.1 g, b) 0.2 g, c) 0.3 g SA content of hydrogel fibers were measured under different tensile strains and different cycle times.


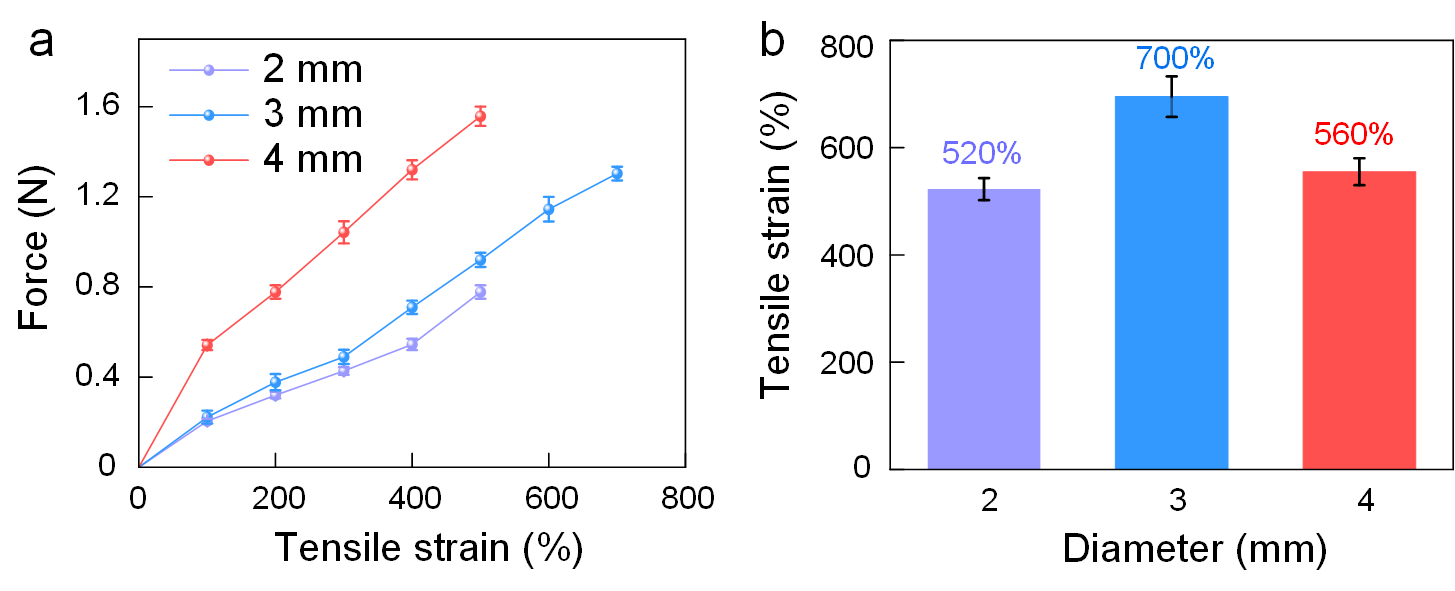


**Figure S6.** Mechanical tensile testing of hydrogel fibers of different diameters. a) Force-tensile curve of hydrogel fibers of different diameters. b) The ultimate tensile strength of fibers of different diameters.


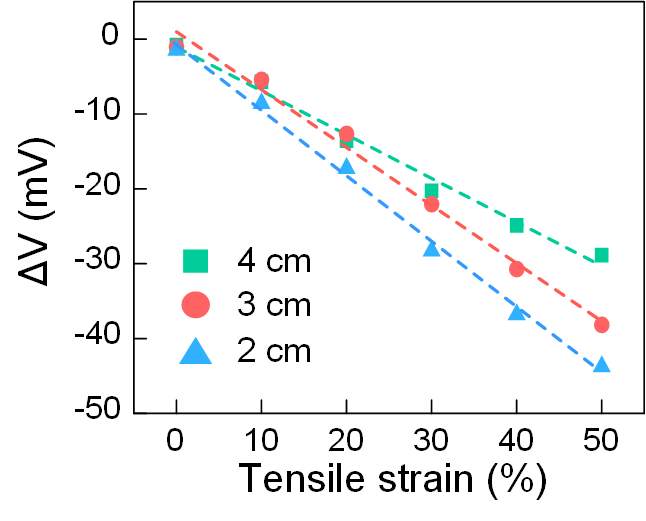


**Figure S7.** Comparison of the HOWS sensor performance of hydrogel fibers with different lengths.


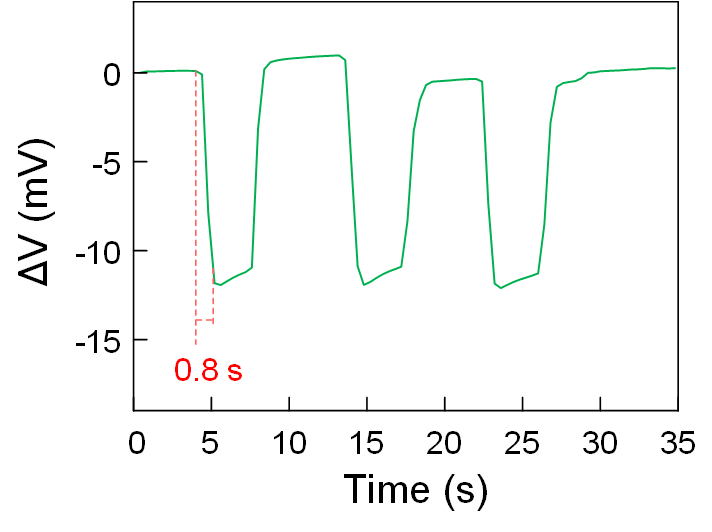


**Figure S8.** The response time of the HOWS sensor.


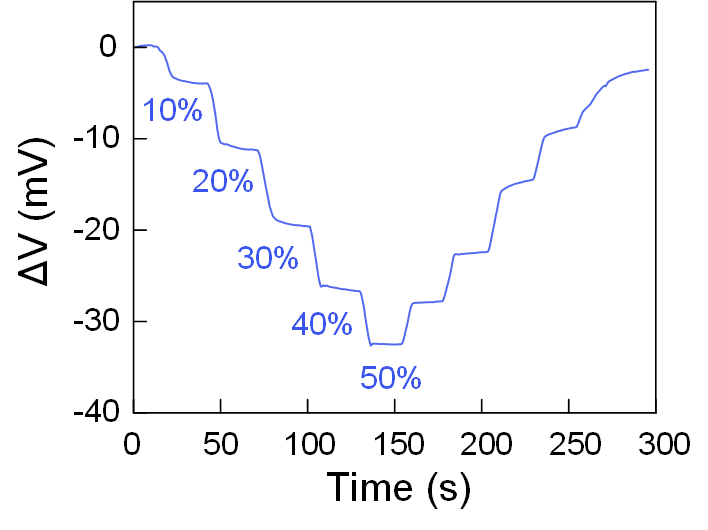


**Figure S9.** The range of HOWS sensors. The HOFS sensor step change curve from 0 to 50%.


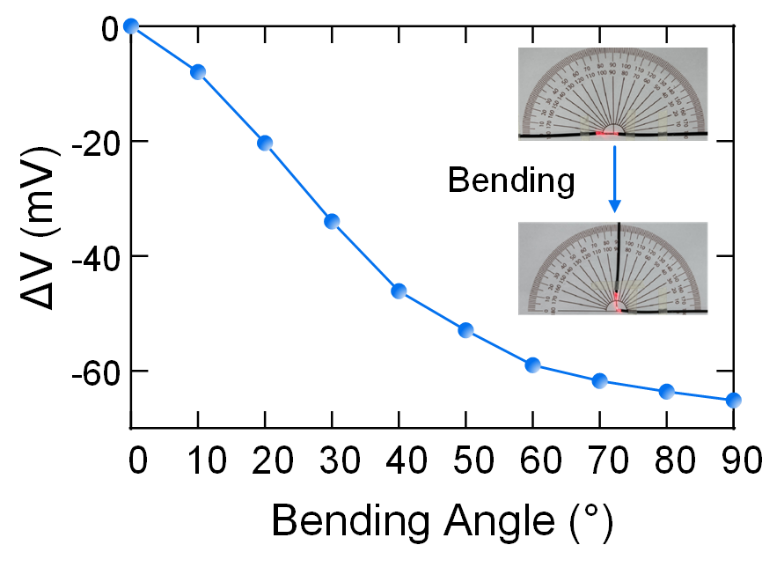


**Figure S10.** Response of the HOWS sensor to bending deformation.


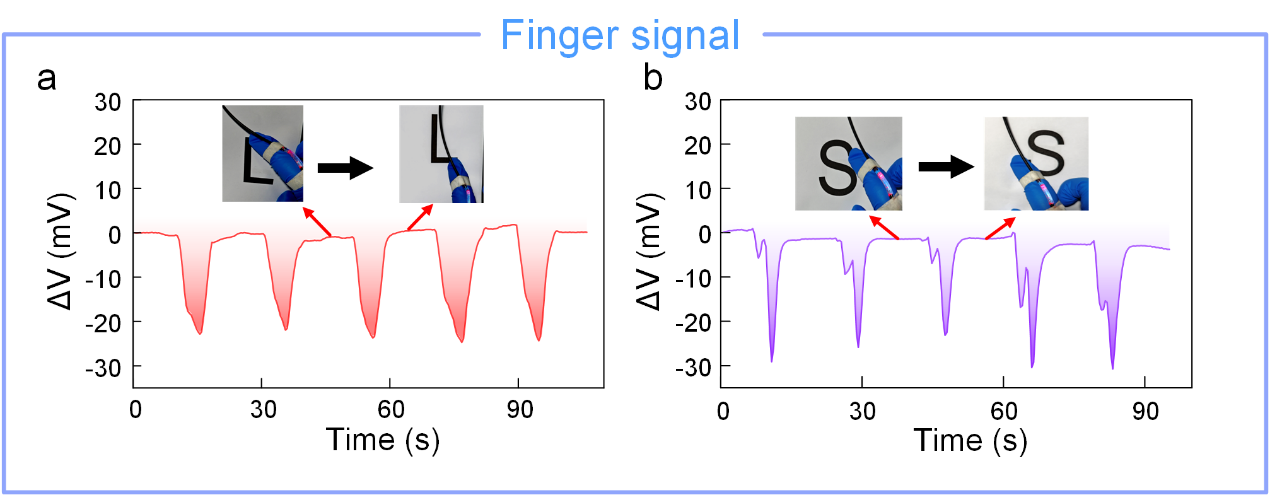


**Figure S11.** Signal test for finger joint writing, including a) letter "L" writing test, and b) letter "S" writing test.


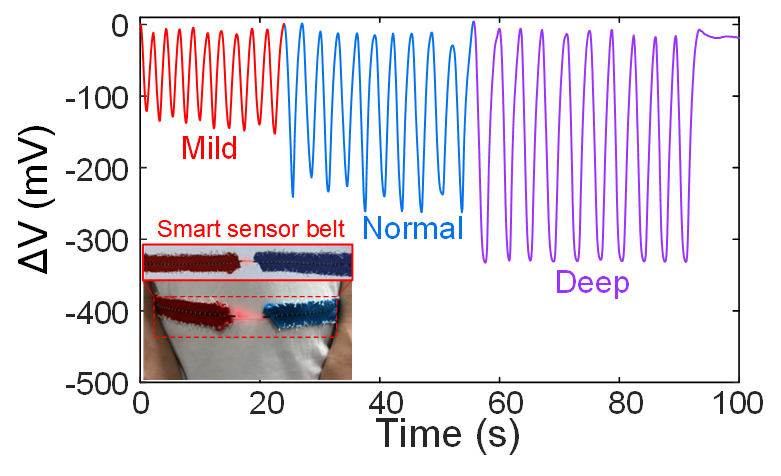


**Figure S12.** Experiment of smart fabric belt based on the HOWS sensor.


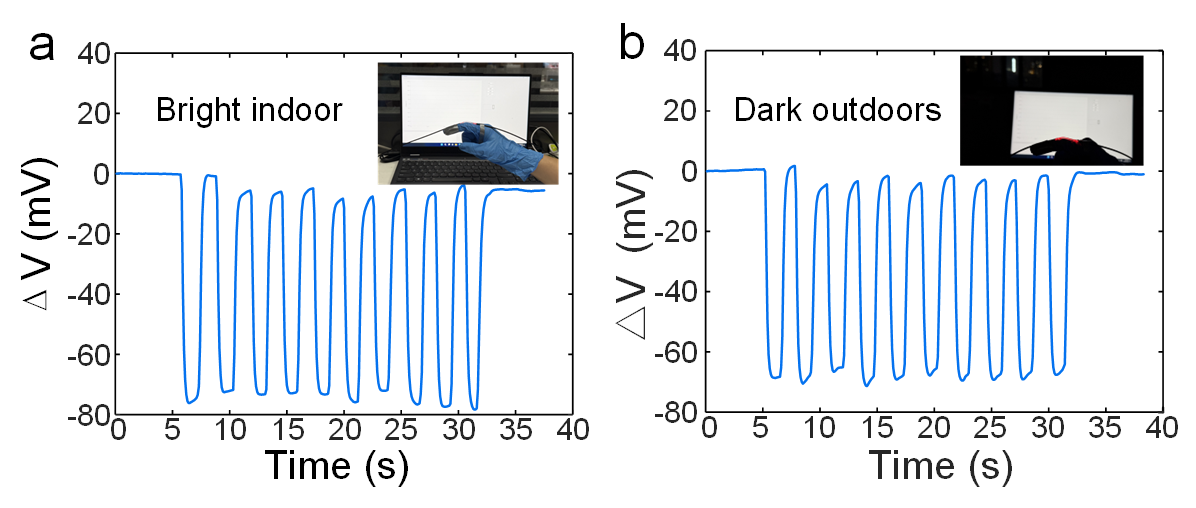


**Figure S13.** Comparison of signal acquisition capabilities of the HOWS sensor in different scenarios. a) Signal changes of the HOWS sensor when the finger is bent 10 times in a bright indoor environment. b) Signal changes of the HOWS sensor when the finger is bent 10 times in a dark outdoor environment.


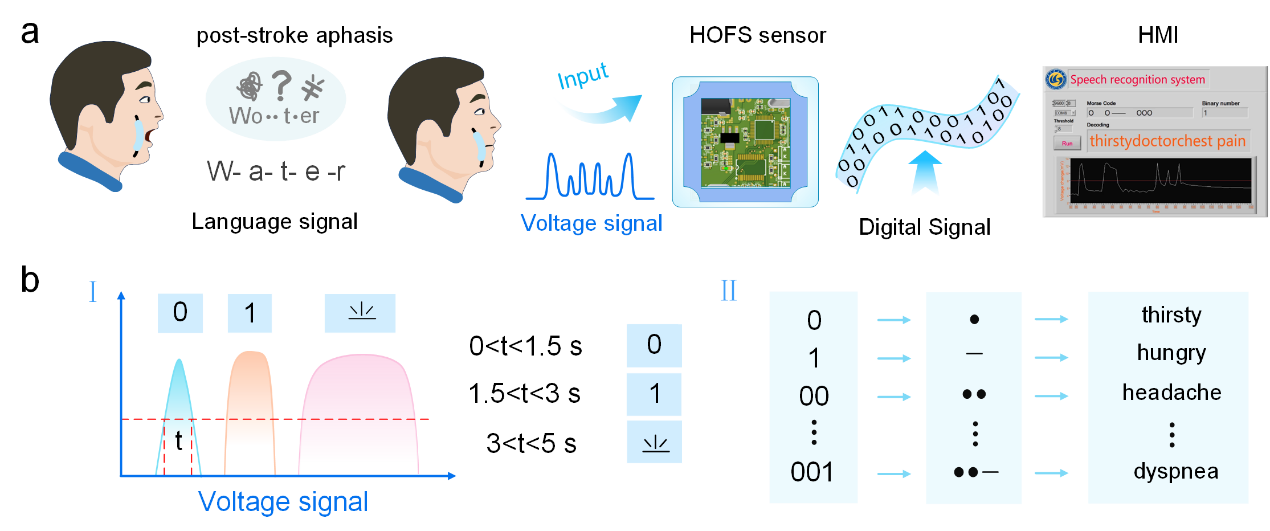


**Figure S14.** Signal transmission process of speech translation system based on masseter signal. a) Masseter signal acquisition process. b) Speech signal decoding rules.


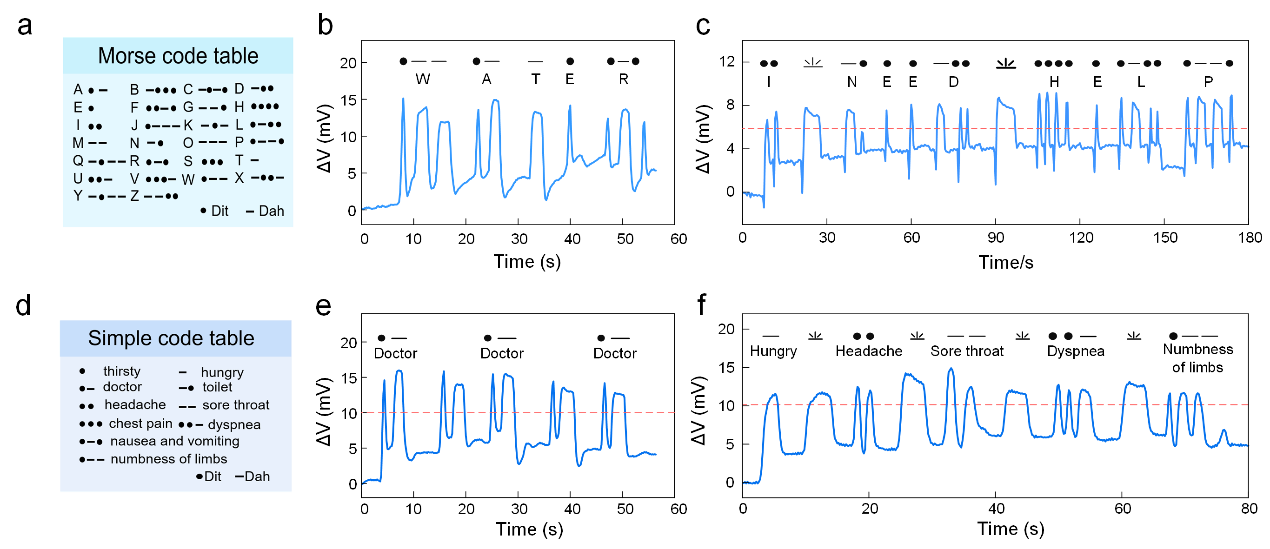


**Figure S15.** Masseter speech translation system test. a) Speech signal decoding table based on Morse code. b) Word translation test. c) Sentence translation test. d)Signal decoding table based on clinical terms. e) Word repetition test. f) Phrase continuity test.


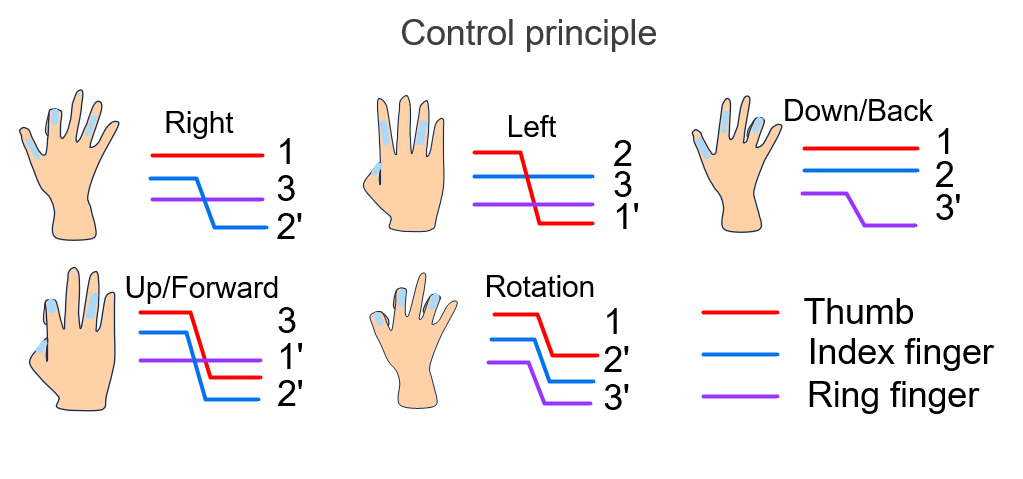


**Figure S16.** The control principles of medical device movement.

**
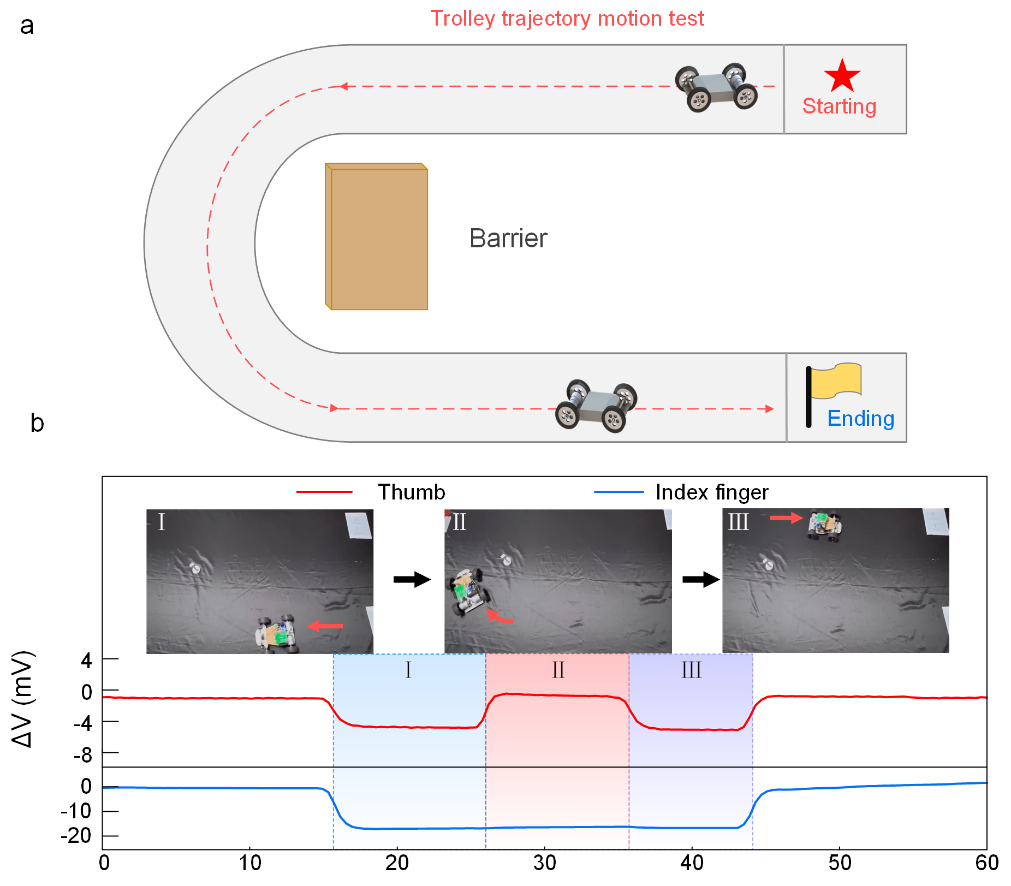
**

**Figure S17.** The trolley trajectory motion test. a) Schematic diagram of the turning trajectory of the trolley. b) The data of HOFS sensors for the trolley trajectory test.

**
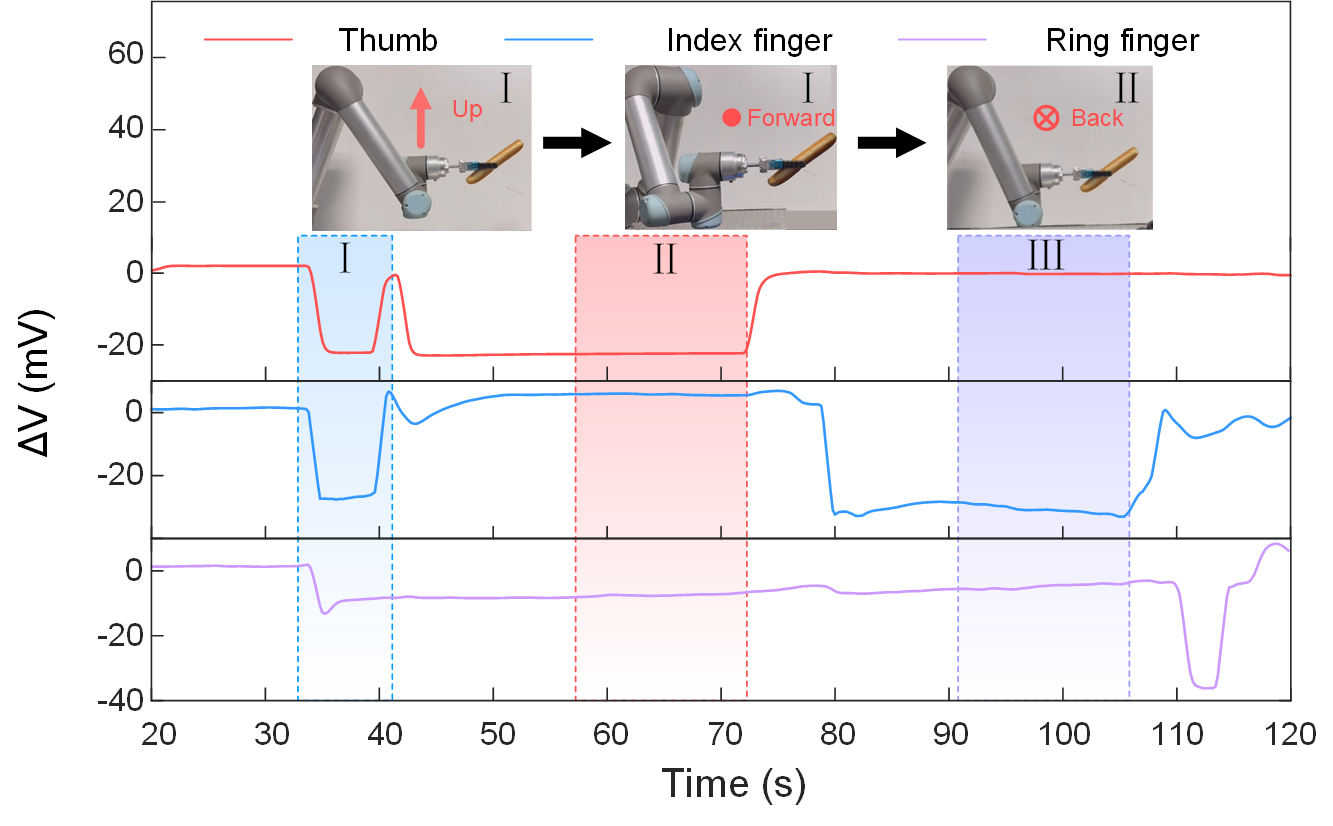
**

**Figure S18.** Food eating aid based on robotic arm movement control.


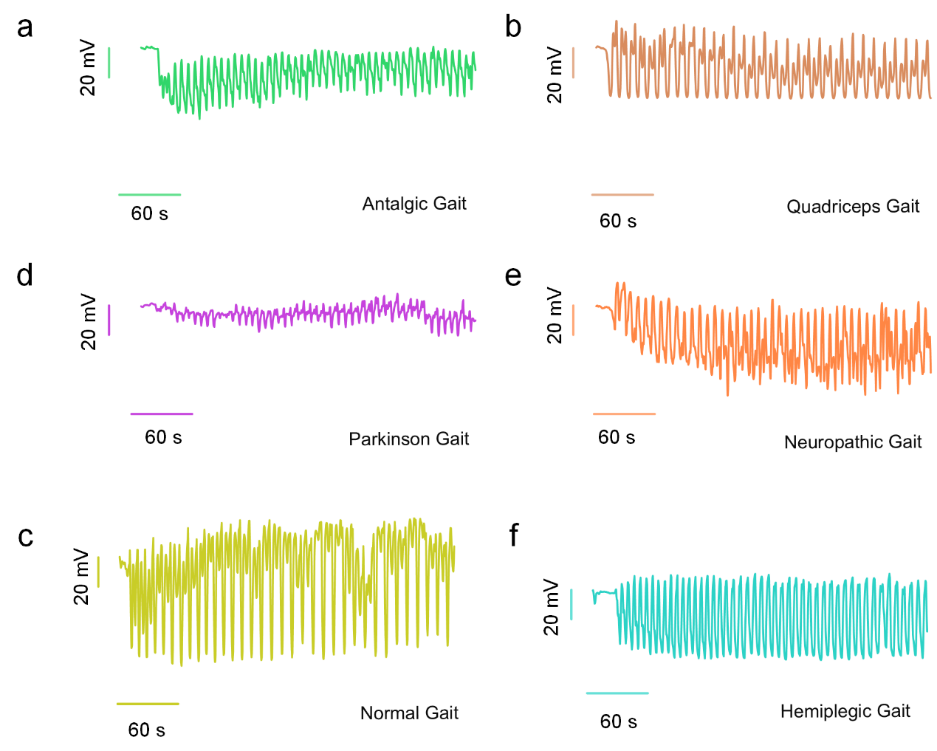


**Figure S19.** Gait time-domain signal characterization plots. a) Antalgic gait signal. b) Quadriceps gait signal. c) Normal gait signal. d) Parkinson’s gait signal. e) Neuropathic gait signal. f) Hemiplegic Gait signal.


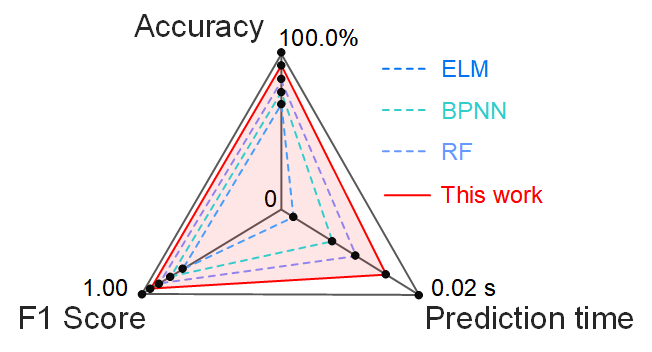


**Figure S20.** Comparison of performance indicators of different data processing algorithms.


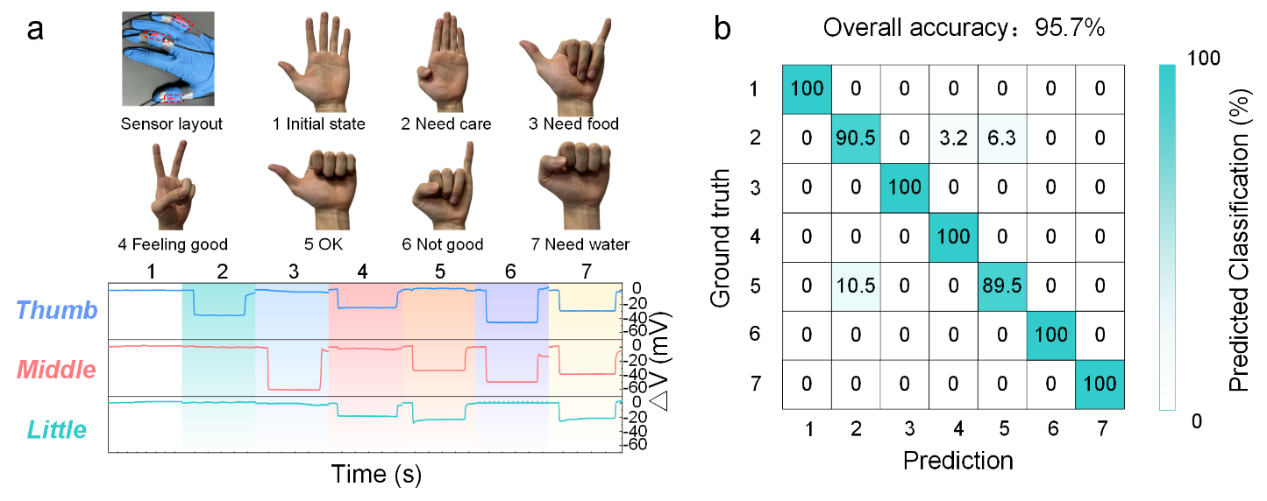


**Figure S21.** Medical gesture recognition experiment. a) The arrangement of HOWS sensors and the information expressed by different medical gestures. b) Confusion matrix for gesture signal classification and recognition results.


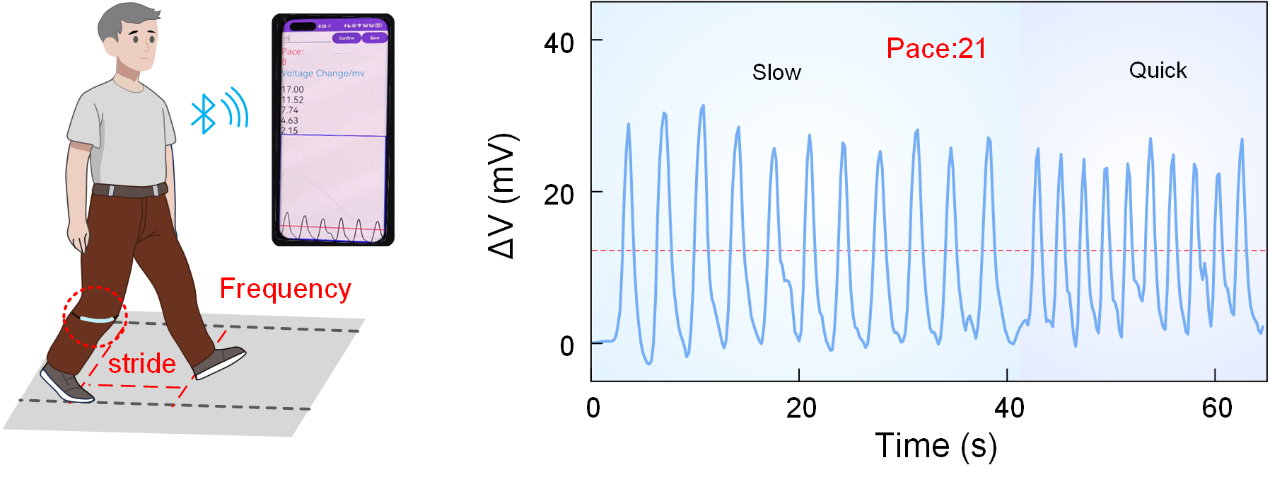


**Figure S22.** The mobile phone health monitoring app used for recording the amount of exercise and exercise curve.
